# Autologous bone marrow stem cell transplantation for the treatment of postoperative hand infection with a skin defect in diabetes mellitus: A case report

**DOI:** 10.3892/ol.2014.1998

**Published:** 2014-03-24

**Authors:** YIHONG LIU, YUCHEN LIU, PUJIE WANG, HAOMING TIAN, JIANZHONG AI, YANGBO LIU, YI ZHOU, ZHONGWEN LIU, WENJUN GUO, SHENKE YANG

**Affiliations:** 1 Department of Endocrinology and Metabolism, West China Hospital, West China Medical School, Sichuan University, Chengdu, Sichuan 610041, P.R. China; 2 Diabetic Centre of Control and Prevention, The People’s Liberation Army 520 Hospital, Mianyang, Sichuan 621000, P.R. China; 3 Core Facility of Genetically Engineered Mice, State Key Laboratory of Biotherapy and Cancer Center, West China Hospital, West China Medical School, Sichuan University, Chengdu, Sichuan 610041, P.R. China

**Keywords:** bone marrow mesenchymal stem cell, autotransplantation, diabetes mellitus, skin defect, tumorigenicity

## Abstract

Among stem cells, autologous mesenchymal stem cells (MSCs) are ideal for transplantation by virtue of limited rejection reactions and marked proliferative ability. This study presents a novel method by which MSCs were harvested from the bone marrow of a patient who presented with severe post-traumatic infection and a non-healing skin defect in the hand, secondary to uncontrolled diabetes mellitus (DM). An autologous MSC suspension was injected into the persistent skin defect after stabilizing the blood glucose level and appropriate infection control. During the course of a regular 18-month postoperative follow-up, the patient exhibited immediate recovery with no transplant-associated complications, as well as no evidence of tumorigenicity. Thus, transplantation of autologous MSCs may play a role in the clinical application of stem cells, particularly for treatment of skin defects following surgery in cases of DM and for those caused by various other traumas.

## Introduction

There are two types of stem cell in the human bone marrow: Hematopoietic stem cells (HSCs) and mesenchymal stem cells (MSCs). HSCs primarily maintain hematopoietic functions, while MSCs have the ability to differentiate into various mesoderm- and neuroectoderm-derived cells. MSCs are multipotent stromal cells with high proliferative ability that can differentiate into a variety of cell types, including vascular endothelial cells, islet cells and hepatocytes, under certain conditions. Autologous MSCs are considered ideal seed cells, since they can be conveniently obtained without ethical issues and are characterized by limited rejection and marked proliferative ability. With the development of cell and tissue engineering, MSCs, as cell sources in cell and gene therapy as well as tissue engineering, are a key area of study (1,2). In 2002, bone marrow HSC transplantation was applied in the treatment of peripheral angiopathy for the first time (3,4). It has been demonstrated in animal experiments that autologous bone marrow mononuclear cells implanted in ischemic limbs can differentiate into vascular endothelial cells to promote local angiogenesis, suggesting that stem cell transplantation can be used for the treatment of ischemic limb diseases and severe skin defects in cases of diabetes mellitus (DM) (5–7).

Skin is an important part of the body in that it maintains homeostasis and acts as a barrier to the invasion of microorganisms and harmful substances. However, various injuries, including trauma, war wounds, and burn and earthquake injuries often cause extensive skin defects, resulting in bacterial invasion and reproduction, which may also induce water and electrolyte disturbance, septic shock, multiple organ dysfunction and mortality. Currently, auto-skin grafting is the main approach to treating extensive burns or skin defects following trauma (8). However, such a method inevitably results in complications through causing trauma to the normal skin. It is therefore urgent that the methods of repairing extensive skin defects are improved.

DM is a chronic metabolic disorder. Difficulty in skin wound healing and skin defects are the most common chronic complications of diabetes. Diabetic foot, as a representative complication, causes disability as a result of amputation. Although numerous hypotheses have been presented, the mechanism of the development of refractory skin wounds under hyperglycemic conditions remains unclear. Oxidative stress (OS) is considered an important common mechanism (9). However, wound healing is a complicated process involving three stages of inflammatory reactions, cell proliferation, tissue maturity and reconstruction. In DM, nonenzymatic glycosylation accelerates, and the level of glycoxidation end-product increases at advanced stages, which interferes with endothelial cell-leukocyte interactions, inhibits the functions of monocytes/macrophages, reduces cytokine secretion capacity and prolongs the duration of infiltration of these cells in the wound. This in itself can lead to a refractory skin defect (10).

As comprehensive therapies, including conventional anti-infection therapy, wound debridement and dressing changes, and glucose-lowering therapy were ineffective for treatment of postoperative wound infection in the hand in the present report, an extended amputation, right hand amputation or local autologous skin transplant for the skin defect was recommended. However, the patient refused these recommendations being concerned that new skin defects would arise as a result of harvesting skin grafts from healthy and intact skin. The patient chose to undergo autologous stem cell transplantation on the understanding that this option would cause minimal injury and pain. Written, informed consent was obtained from the patient prior to autologous bone marrow MSC transplantation on the skin defect of the right hand in August, 2010. The clinical efficacy and complications, including transplantation-associated rejection reactions and tumorigenicity of transplanted autologous bone marrow MSCs, were investigated for a period of 18 months. This program was approved by the Scientific and Ethics Committee of Sichuan University (Chengdu, China).

## Case report

### Case summary

A 64-year-old male farmer was admitted to the Department of Hand and Foot Surgery at the People’s Liberation Army 520 Hospital (Mianyang, China) on July 22nd, 2010 complaining of elevated blood glucose levels for three years and right hand pain and swelling following trauma for longer than half a month. The patient was diagnosed with DM at a local hospital three years previously, but had not adhered to regular treatment due to personal financial problems. Therefore, blood glucose levels had remained largely unchecked. Notes from a previous hospital visit indicated that the random blood glucose level was 16.08 mmol/l and percentage glycosylated hemoglobin was 12.7%. Approximately half a month previously, the patient had accidentally cut his right hand at work, producing a non-healing wound resulting in the back of the right hand becoming swollen and painful, together with infection, necrosis and a large defect in the skin of the root of the right forefinger. Debridement and sutures at a local hospital achieved unsatisfactory results, and the case was transferred to the People’s Liberation Army 520 Hospital. On admission, a 5.0×3.0 cm-deep skin defect in the metacarpophalangeal joint of the forefinger and back of the right hand back with flavescent fishy effusions was observed. The bone was exposed and visible through the defect, the tendon was ruptured and the distal tendon was pale. Poor local blood supply was observed, and the patient was suffering from local numbness. The defect was irregular and the contusion was severely contaminated. The case was preliminarily diagnosed as wound infection following trauma, vascular, nerve and tendon injuries and complex tissue defects in the right hand, and DM and diabetic nephropathy. Following intensive insulin pump therapy, anti-infection therapy and local wound debridement and dressing changes for one week, blood glucose became well controlled (Table I) but the local wound did not improve. Therefore, amputation of the right forefinger was performed on August 7th, 2010. Following surgery, the surgical wound in the right hand developed suppuration and infection and was poorly healed, with a size of ~5.0×3.0 cm. As the condition had not improved following comprehensive therapy, including anti-infection therapy, wound dressing changes and glucose-lowering therapy, the case was transferred the Diabetic Centre of Control and Prevention for further treatment. After group consultation and discussion, a choice of extended amputation, right hand amputation or local autologous skin grafting was recommended. However, as discussed previously, the patient refused these recommendations and instead opted for autologous MSC transplantation. On August 5th, 2010, patient bone marrow was harvested under local anesthesia and MSCs were separated, cultured and amplified *in vitro* for 14 days. The third generation of MSCs was prepared as a stem cell suspension with a concentration of 6×10^6^ cells/ml. On August 19th, 2010, autologous bone marrow MSC transplantation was performed after the patient provided written, informed consent. No common complications or adverse reactions associated with transplantation were observed. On day 10 after surgery, the patient was completely healed and was discharged from hospital. Before and after autologous stem cell transplantation, blood glucose levels had almost reached the standard range (Table I).

### Reagents

Lymphocyte separation medium was purchased from Shanghai Qcbio Science & Technologies Co., Ltd. (Shanghai, China). Xylene and hematoxylin and eosin (H&E) stain were purchased from Shanghai Source Leaf Biological Technology Co., Ltd. (Shanghai, China). Complete Dulbecco’s modified Eagle’s medium and fetal bovine serum were purchased from Invitrogen Life Technologies (Carlsbad, CA, USA). Penicillin and streptomycin were purchased from Nanjing KeyGEN Biotech Co., Ltd. (Nanjing, China).

### Collection of autologous bone marrow

Following completion of pre-operative examinations, the patient fasted on the morning of the surgery. The patient was placed in a prone position and conventional disinfection and sterilization procedures were followed. Blood bags and storage solution were prepared. Bone marrow was collected from the bilateral posterior superior iliac spine under local anesthesia. Approximately 10 ml bone marrow was collected from each of three sites at 1-cm intervals along the lateral superior edge of the ilium (a total of 30 ml bone marrow).

### Separation of bone marrow MSCs

The collected bone marrow samples were diluted with twice the volume of physiological saline on a laminar flow cabinet, and the resulting suspension was transferred to a lymphocyte separation medium (at a ratio of 2:1). The obtained solution was centrifuged at 640–800 × g for 20 min at 20°C. The white membrane layer in the 15 ml centrifuge tube was gently transferred to a clean Eppendorf tube, washed twice with double the volume of phosphate buffer solution (pH 7.2–7.4), and centrifuged at 450 × g for 10 min. Mononuclear cells were separated, amplified *in vitro* for 14 days and passaged to the third generation. The precipitate was diluted with 30 ml physiological saline to produce a stem cell suspension containing 6×10^6^ cells/ml. Of this suspension, 1 *μ*l was used to count cells and observe the cell morphology under a microscope.

### Autologous bone marrow MSC transplantation

Autologous bone marrow MSC transplantation was performed under strictly aseptic conditions in the operating room. The infection site on the right hand was routinely debrided and the MSC suspension was transplanted into the wound by injection of 0.3–0.5 ml in each site at 1.0-cm intervals. The injection depth was 0.5–1.0 cm. Following injection, the wound was protected with sterile gauzes and bandages.

### Observation of wound healing

The wound surface of the skin defect prior to surgery and during recovery was recorded using a digital camera, and any presence of rejection reactions, hand inflammation and swelling was recorded.

### H&E staining

Skin tissues were surgically collected, fixed in 4% paraformaldehyde overnight, dehydrated with a series of ethanol solutions, embedded in paraffin and cut into sections. The sections were deparaffinized with xylene, rehydrated, stained with H&E stain and enveloped. The pathological changes of the skin were observed using the Olympus BX41 microscope (Olympus Corporation, Tokyo, Japan).

### Observations following transplantation

**Figure 1:**
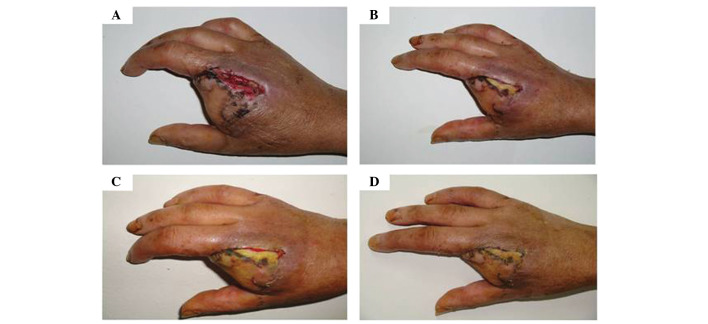
Transplantation sites before and after surgery showing significant improvement in swelling and inflammation. (A) Prior to surgery, the wound was 5.0×3.0 cm in size; (B) 48 h after surgery, the wound was 4.0×1.0 cm in size; (C) 96 h after surgery, the wound was 4.0×0.5 cm in size; and (D) 192 h after surgery, the wound had healed.

The pain, coldness and numbness in the right hand improved 24 h after transplantation. After 48 h, the purulent secretions from the wound had clearly decreased, local swelling alleviated, local ischemia gradually improved and the wound shrank from 5.0×3.0 cm to 4.0×1.0 cm (Fig. 1A and B). After 96 h, the wound was 4.0×0.5 cm in size (Fig. 1C) and local ischemia gradually improved. After 192 h, the wound had completely healed (Fig. 1D). Therefore, following autologous bone marrow MSC transplantation, the injection puncture wounds healed rapidly. No local abnormal symptoms or signs were observed, and blood, urine and stool tests, and biochemical examinations (including liver and renal function tests) revealed no systemic abnormalities.

**Figure 2:**
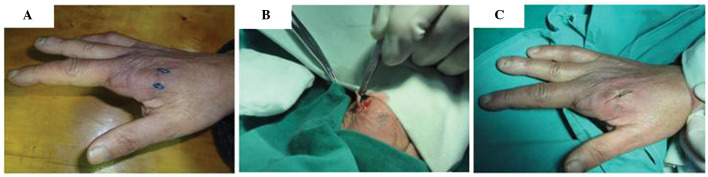
Histological examination of the skin (18 months post-surgery). (A) The site on local healed skin where tissues are sampled for biopsy; (B) local skin is sampled for biopsy; and (C) biopsy site sutured after tissue sampling.

**Figure 3:**
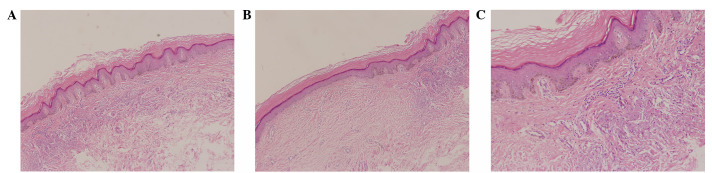
Histopathological examination of hematoxylin and eosin-stained skin from the healed wound. (A) Normal skin (magnification, ×100); (B) mild epidermal hyperplasia with hyperkeratosis and increased pigment in local basal cells (magnification, ×100); and (C) mild epidermal hyperplasia with hyperkeratosis, hyperplasia and degeneration of collagen fibers, and minor infiltration of lymphocytes in the small perivascular regions (magnification, ×200).

The patient was readmitted to the People’s Liberation Army 520 Hospital for examination 18 months after surgery. The local condition of the hand that underwent autologous bone marrow MSC transplantation was stable and no transplantation-associated rejection reactions had occurred. A sample of local full-thickness skin tissue, at a size of 0.7×0.3×0.2 cm, was cut from the healed wound (Fig. 2) for pathological examination. H&E staining revealed mild epidermal hyperplasia with hyperkeratosis, increased pigment in local basal cells, hyperplasia and degeneration of collagen fibers, and minor infiltration of lymphocytes in the small perivascular regions of the superficial dermis. These observations are not significantly different from the pathology of normal skin tissues. In addition, there was no evidence of tumor formation (Fig. 3).

## Discussion

It has been demonstrated that skin constructed by MSCs used as seed cells can significantly promote healing of skin defects. Such wounds possess full-thickness skin structures following repair (11–13). During the process of wound healing, MSCs are closely involved in the formation of small blood vessels in the granulation tissue (14). MSCs can differentiate into vascular endothelial cells and are involved in repair of the skin defect in the wound micro-environment. It has been reported that MSCs may also be used for treatment of ischemic diseases (15–18). In a study by Subrammaniyan *et al* (19), injection of bone marrow-derived mononuclear cells had satisfactory efficacy in the treatment of six cases of DM with critical limb ischemia and skin defects, and amputation was avoided in all patients undergoing autologous bone marrow-derived mononuclear cell injection.

However, it is debated whether MSCs transplanted into the body may induce various gene mutations resulting in infinite cell proliferation and growth similar to tumor cells or even induce tumorigenesis (20–22). It has been demonstrated that embryonic stem cells (ESCs) isolated from rodents and humans are very similar to embryonal carcinoma cells, and their potent tumorigenicity has the potential to lead to teratomas (23). Stem cell tumorigenicity is the key obstacle to the safe use of stem cell-based regenerative therapies. Although certain adult stem cell therapies appear to be safe, they have only a narrow range of application in human disease. Human induced pluripotent stem cells are predicted to possess tumorigenic potential equal to or greater than that of ESCs (23).

Based on the aforementioned issues surrounding stem cell tumorigenicity, further follow-up observation and clinical study were conducted for the present patient. Pathological examinations of the full-thickness skin tissues from the healed wound revealed no significant difference from the pathology of the normal skin tissue, and no tumor formation was identified (Fig. 3). Thus, we hypothesize that autologous transplantation of MSCs amplified *in vitro* may be a novel, simple and effective approach to the treatment of severe skin defects and infection. In addition, such therapy presents a solution to the problems of severe wound infection and poor local blood supply in DM without transplantation-associated rejection and tumor formation, thereby achieving the goals of treatment. In addition, the present study provides a novel method for the treatment of other skin defects caused by various traumas, including burns, knife wounds and earthquake injuries, using autologous MSC transplantation in clinical practice.

The identification and study of stem cells is a promising field in biomedicine. However, it has been reported that the tissues grown from skin-derived autologous stem cells may still be rejected by the immune system (24). Consequently, the application of autologous MSC transplantation in clinical treatment requires further studies, and there is a great need to investigate transplant-associated rejection reactions, tumorigenicity and the long-term efficacy of autologous MSC transplantation. The methodology of autologous MSC transplantation in the treatment of skin defects induced by various traumas should be improved, and high-quality, multicenter, randomized, double-blind and placebo-controlled trials are required to demonstrate the clinical efficacy of this novel therapy. In addition, certain aspects in particular should be noted. Firstly, the dose of MSCs, observation duration and data units should be standardized and unified, and a widely recognized criteria for assessment of therapeutic efficacy should be employed as far as possible. Additionally, besides the observation of the size of the wound, it is also important that the expanded MSCs be labeled with bromodeoxyuridine (BrdU) prior to transplantation. Full-thickness skin from the wound and the healed skin should be incised between two and 12 weeks after surgery for H&E staining and BrdU immunohistochemistry to pathologically compare the two. Furthermore, observation reports of adverse reactions and tumorigenesis should be normalized and standardized, and the follow-up period should be extended so that the long-term efficacy may be assessed. In addition, the negative results of the clinical trials should be emphasized and more attention to the ethical issues concerning stem cell therapy should be paid. Finally, the differentiation mechanism and induction conditions of MSCs, and the mechanism underlying their efficacy in the treatment of skin defects, remain unclear (13). It is speculated that MSCs can differentiate into vascular endothelial cells in a wound micro-environment and be further involved in wound repair and promote angiogenesis (25). Additional animal experiments and basic studies are required to observe the transition of MSCs to endothelial cells in local transplantation regions, the release of multiple cytokines in the local region and signal transduction (26).

The novel therapy presented in the current study may solve the current problems of severe wound infection and poor local blood supply in DM, without transplantation-related rejection reactions and tumor formation, thereby achieving the goals of treatment.

## Figures and Tables

**Table I:** Blood glucose levels during two hospital admissions.

		Blood glucose level, mmol/l				
		
Date	Diabetes mellitus treatment protocol	Prior to breakfast	2 h after breakfast	2 h after lunch	2 h after supper	2:00 AM
2010-07-29^a^	Intensive insulin pump therapy (Humalog at 63 U/day)	5.2	8.5	8.7	7.1	5.2
2010-08-12^a^	Humalog Mix 50 (16, 12 and 14 units)	6.1	5.2	10.5	7.8	8.8
2010-08-19^b^	Humalog Mix 50 (16, 13 and 14 units)	4.8	10.4	8.8	8.7	8.8
2010-08-20^c^	Humalog Mix 50 (16, 12 and 14 units)	5.7	6.7	8.1	9.3	5.5
2010-09-02^d^	Humalog Mix 50 (16, 14 and 14 units)	5.7	8.1	7.9	7.9	7.1
2012-02-29^e^	Metformin (500 mg, 3 times/day)	7.0	16.4	12.7	11.8	Unmeasured
2012-03-05^e^	Metformin (850 mg, 3 times/day)	6.1	10.0	8.1	9.1	8.9

a Prior to stem cell therapy;

b during stem cell therapy;

c following stem cell therapy;

d following first discharge from hospital; and

e during second hospital admission.
